# The Neurobiology of Time Processing

**DOI:** 10.1155/2016/1706373

**Published:** 2016-06-01

**Authors:** Francesca Frassinetti, Marinella Cappelletti, Domenica Bueti

**Affiliations:** ^1^Department of Psychology, University of Bologna, 40127 Bologna, Italy; ^2^Fondazione Salvatore Maugeri Hospital IRCCS, 46042 Castel Goffredo, Italy; ^3^Institute of Cognitive Neuroscience, University College London, London WC1N 3AR, UK; ^4^Department of Psychology, Goldsmiths College, University of London, London SE14 6NW, UK; ^5^Biomedical Imaging Research Center (CIBM) and École Polytechnique Fédérale de Lausanne (EPFL), 1015 Lausanne, Switzerland; ^6^International School for Advanced Studies (SISSA), 34136 Trieste, Italy

Time is the most elusive dimension of everyday experiences; we cannot “see” or “touch” time; nevertheless we can sense and represent its passage over a wide range of temporal scales, from a few hundreds of milliseconds to years. For example, we are able to appreciate and reproduce time in the millisecond range while playing music and we can perceive the passage of a few minutes of time while waiting for our train to arrive. We can also remember how many years back we first met our best friend or estimate how many minutes are ahead of our next meeting.

However, time does not only span across different scales; it can also be used for different purposes. We can sense the passage of time either to judge how long it takes to commute from and to work (i.e., duration estimation) or to establish which runner reached first the finish line (i.e., temporal order judgments). We can also use time to predict when a traffic light is likely to turn from red to green (i.e., temporal expectations), and we can place ourselves in a temporal prospective by remembering our past and imagining our future (i.e., mental time travel).

In the last two decades, the majority of investigations in the field of temporal cognition focused on the identification and the role of brain regions associated with time representation and processing. All these neuropsychological, neuroimaging, and brain stimulation studies in humans together with electrophysiological works in animals show that several brain areas, including basal ganglia, cerebellum, and posterior parietal, premotor, prefrontal, and insular cortices, are engaged in temporal computations [1–3]. Although the role of each of these “putative” time regions is unknown, it is becoming increasingly clear that time in different scales and the use of time for different purposes are associated with different temporal networks and subserved by distinct temporal mechanisms.

In this special issue, we are very pleased to present a series of articles investigating distinct aspects of the perception of time. The first novelty of this issue is to collect papers exploring different duration ranges (from milliseconds to seconds to minutes) by using different methodological approaches (psychophysics, neuropsychology, brain stimulation, and neuroimaging) and different temporal tasks (temporal order judgments, temporal predictions, temporal reproduction and discrimination, and mental time travel). Specifically, two studies focus on temporal predictions, that is, the capacity of estimating time in order to predict and anticipate the occurrence of behaviorally relevant events. In the first of these studies, P. Filip et al. in “Neural Network of Predictive Motor Timing in the Context of Gender Differences” explore gender differences in the neural networks associated with millisecond time estimation used to perform a visually guided action. The results highlight the pivotal role of the cerebellum in temporal predictions and provide novel insights into our understanding of gender differences linked to time processing. In the second of these works, Y.-H Su and E. Salazar-López in “Visual Timing of Structured Dance Movements Resembles Auditory Rhythm Perception” investigated whether similar perceptual mechanisms as for auditory rhythms were employed when observing temporally structured dance movements. In two experiments, using visual and auditory stimuli, respectively, parallel results emerged, suggesting similarities between auditory and visual rhythmic timing.

Investigations on temporal predictions were complemented by two other studies exploring the neural basis of temporal judgments in the seconds range. M. Riemer at al. in “Systematic Underreproduction of Time Is Independent of Judgment Certainty” show that the posterior parietal cortex (PPC) is involved in mediating temporal discrimination but not temporal reproduction judgments. Using stimuli of a similar duration range, I. Patanè et al. in “Prismatic Adaptation Induces Plastic Changes onto Spatial and Temporal Domains in Near and Far Space” explore the effect of prismatic adaptation on both spatial and temporal judgments and investigate how temporal judgments are influenced by spatial manipulations (i.e., estimating time in near and far space).

From millisecond to much longer temporal ranges, two articles concern metal time travel (MTT), namely, the human ability to travel mentally backwards and forwards in time in order to reexperience past events and preexperience future ones. These studies explored different aspects of MTT. F. Anelli et al. in “Age-Related Effects on Future Mental Time Travel” explored the potential changes of MTT in aging. By behaviorally testing young and aging adults, the authors explored two different components of MTT, self-projection, that is, the ability to project the self towards a past or a future location, and self-reference, that is, the ability to determine whether events are located in the past or in the future with respect to a self-location. The study by C. Ansuini et al. in “The Role of Perspective in Mental Time Travel” asks whether people can adopt the temporal perspective of another person when travelling through time. To elucidate similarities and differences between time travelling from one's own perspective or from the perspective of another person, participants are asked to mentally project themselves or someone else (i.e., a coexperimenter) to different time points. Following a similar rationale of time travelling, M. Bonato et al. in “Hemispatial Neglect Shows That “Before” Is “Left”” investigate the ability of right brain damaged patients, with and without hemispatial neglect, to categorize events of a story as occurring before or after a central event. The results show that the event occurring immediately before the reference leads to particularly slow responses in neglect patients.

In sum, this special issue presents several novel and previously unexplored issues related to time processing, such as the effect of gender and age, the multimodality of time processing, as in the context of space, and the role of perspective (self/other) in the mental travel of time. Together, these original issues provide a new and interesting overview on the advances in the field of temporal cognition.

*Francesca Frassinetti* *Francesca Frassinetti* *Marinella Cappelletti* *Marinella Cappelletti* *Domenica Bueti* *Domenica Bueti*
